# Compositional profiling of volatile and non-volatile compounds in Australian cocoa nibs: Insights into origin-dependent variability

**DOI:** 10.1016/j.fochx.2026.104220

**Published:** 2026-07-16

**Authors:** Jia Wang, Marlize Zaretha Bekker

**Affiliations:** School of Agriculture and Food Sustainability, The University of Queensland, St Lucia, QLD 4072, Australia

**Keywords:** Cocoa nibs, Australian cocoa, Volatile compounds, Phenolic compounds, HS-SPME-GC–MS, Food composition

## Abstract

Chemical composition plays a central role in determining flavour potential and provenance-based differentiation of cocoa, however, compositional data for emerging production regions such as Australia remain scarce. This study provides a comprehensive compositional dataset for Australian cocoa nibs sourced from three producers in Far North Queensland. Nibs were analysed for organic acids, total phenolic and flavonoid contents, antioxidant capacity, phenolic compounds and methylxanthines, and volatile profiles using HS-SPME-GC–MS and significant producer-dependent differences were observed. Nibs fermented at the same facility showed greater similarity in volatile profiles than nibs derived from the same genotype but fermented at different sites using the same protocol. Australian cocoa nibs exhibited chemical profiles comparable to those reported for established cocoa-producing regions, while retaining producer-specific compositional signatures. These results provide foundational compositional data for Australian-grown cocoa and shows the role that fermentation environment may play in shaping flavour-relevant chemistry, supporting provenance-based differentiation within emerging cocoa industries.

## Highlights

• Australian cacao nibs showed non-volatile and volatile profiles comparable globally.
• TPC, TFC and epicatechin varied between producers under standardised processing.
• Lactic-to-acetic acid ratios reflect site-specific fermentation outcomes.
• HS-SPME-GC–MS revealed variation in aroma-active compounds between producers.
• Multivariate analysis grouped nibs by fermentation site rather than genotype.

## Graphical abstract

**Figure:**
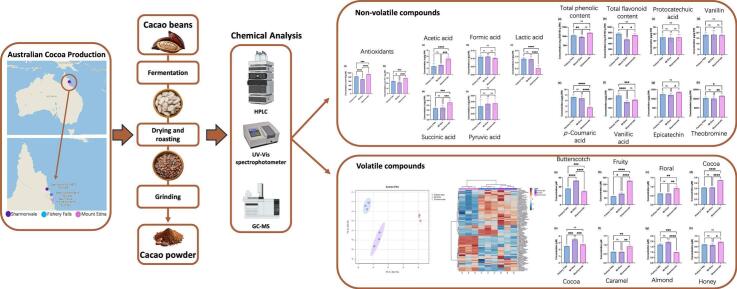

## 1 Introduction

Cocoa (*Theobroma cacao L*.) is a globally important crop whose chemical composition underpins flavour quality and origin differentiation in chocolate. Its botanical name, derived from Greek, reflects its long-standing cultural and sensory significance as the “food of the gods” (Montagna et al., 2019). Global cocoa production is dominated by smallholder farming systems, supplying approximately 90% of global output, with 70% of this supply originating from Ghana and Côte d'Ivoire in West Africa (Kongor et al., 2024; Roth, 2022; Wessel & Quist-Wessel, 2015). Additional major cocoa-producing countries include Indonesia, Nigeria, Cameroon, Ecuador, and Colombia, Papua New Guinea (PNG), and Brazil (Etaware, 2022; Roth, 2022). Cocoa production is largely confined to tropical regions near the equator, where rainfall throughout the year supports growth; however, production remains vulnerable to climate variability, pest and disease pressure, and socio-economic constraints (Anning et al., 2022; Scudder et al., 2022). In recent years, concerns surrounding deforestation, labour practices, and long-term sustainability of smallholder-based system have increased, leading to greater interest in alternative cocoa production models and alternative cocoa origins that emphasise quality, traceability and provenance (Perez et al., 2021).

Australia represents a small but developing cocoa-production region. Over the past two decades, cocoa cultivation has expanded, with production concentrated in Far North Queensland across a limited number of growers operating small-scale or mixed farming systems (Diczbalis, 2013; Roth, 2022). Although the total production volume remains low, Australian cocoa is primarily positioned within premium and craft markets (Roth, 2022). Ongoing research and experimentation, including investigation into irrigated production in marginal environments, suggests potential for further expansion and diversification of cocoa production systems under Australian conditions (Diczbalis et al., 2010; Roth, 2022). Given increasing challenges facing global cocoa production, including climate change, disease pressure, and supply constraints, understanding the quality potential of emerging cocoa-producing regions may become increasingly important for future supply diversification (Anning et al., 2022; Roth, 2022; Scudder et al., 2022).

Cocoa flavour is a primary determinant of commercial quality, particularly for “fine/flavour” cocoa, which is characterised by desirable sensory notes (e.g., fruity, floral, herbal, woody, nutty, caramel-like, and chocolate base notes) but still lacks universally accepted objective chemical criteria that clearly discriminate it from bulk cocoa (Ullrich et al., 2023). The chemical complexity of cocoa underpins its wide diversity of flavour expression and forms the basis for classification into bulk and fine/flavour categories (Asiedu, 2024). More than 600 volatile and semi-volatile compounds have been reported in cacao beans and cocoa products, spanning chemical classes such as aldehydes, pyrazines, esters, alcohols, acids, furans, and lactones (Kongor et al., 2016). In addition to these aroma-active compounds, non-volatile constituents, including polyphenols, methylxanthines, and organic acids, are associated with bitterness, astringency, acidity, and mouthfeel, thereby has the potential to contribute to perceived quality (Belitz et al., 2009). Cocoa sensory properties arise from the interaction between these volatile and non-volatile compounds, the composition of which is defined by both pre-harvest and post-harvest conditions.

Genetic background strongly influences cocoa flavour potential, with major cacao varieties exhibiting distinct sensory tendencies. Forastero cacao, which represents approximately 90% of global production, is typically associated with robust cocoa and roasted notes and is widely used for mass-market chocolate manufacture (Fowler & Beckett, 2009). In contrast, Criollo and Trinitario varieties are frequently classified as fine/flavour cocoas due to their more nuanced sensory profiles, including floral, nutty, earthy, and alcoholic notes (Jahurul et al., 2013; Rusconi & Conti, 2010; Wood & Lass, 2008). Other genetic groups, such as Nacional cacao from Ecuador, are similarly renowned for distinctive fruity and aromatic characteristics (Afoakwa et al., 2008). It is important to recognise that the expression of these genetically driven flavour traits is strongly modulated by terroir and post-harvest practices (Manhounou et al., 2024). For example, Forastero beans subjected to carefully controlled fermentation protocols can yield flavour profiles comparable to fine/flavour cocoa, while suboptimal processing of fine-flavour genotypes can result in bulk-like sensory outcomes (Colonges et al., 2022; Hinneh et al., 2020). Understanding how these compositional factors manifest in emerging cocoa origins is critical for positioning such products within fine/flavour markets.

Post-harvest processes such as fermentation, drying, and roasting play a significant role in flavour and quality expression (Urbańska et al., 2019). Fermentation drives microbial metabolism that generates ethanol and organic acids and promotes the formation of flavour precursors, including amino acids, peptides, and reducing sugars, while also modulating acidity and astringency (Belitz et al., 2009; Hamdouche et al., 2017; Misnawi et al., 2002). Organic acids, particularly citric, lactic, and acetic acids, undergo significant changes in pulp, which influences bean pH and enzyme activity relevant to flavour precursor availability (Belitz et al., 2009; Ho et al., 2014). During roasting, Maillard and Strecker pathways convert these precursors into key odorants, including pyrazines and aldehydes that contribute cocoa, roasted, nutty, and praline-like notes (Frauendorfer & Schieberle, 2006; Oracz & Nebesny, 2019; Rizzi, 1999; Afoakwa et al., 2008). In parallel, phenolic compounds such as (−)-epicatechin, catechin, procyanidins, and phenolic acids contribute to bitterness and/or astringency and underpin antioxidant capacity commonly captured via total phenolics/total flavonoids assays (Afoakwa et al., 2008; Hermund et al., 2025; Jalil & Ismail, 2008; Rojas et al., 2022).

Despite increasing interest in Australian cocoa production, compositional data describing volatile and non-volatile constituents of Australian-grown cocoa remains limited. Consequently, the extent to which producer-level differences are reflected in flavour-relevant chemistry remains poorly understood. This study addresses this knowledge gap through the integrated characterisation of phenolic compounds, methylxanthines, organic acids, antioxidant capacity, and volatile composition in cocoa nibs from three Far North Queensland origins. Sector analysis highlights that terroir (microclimate/soil), and in particular, fermentation environment can strongly influence flavour development and may provide opportunities for origin-linked differentiation (Roth, 2022). This study aims to evaluate producer-level variation under broadly aligned commercial post-harvest conditions and to explore potential contributions of fermentation environment and genetic background to compositional variation in Australia cocoa systems. While post-harvest processing conditions were broadly aligned, variation in genotype, drying location, and uncharacterised microbial communities associated with spontaneous fermentation represent potential sources of variability that could not be experimentally disentangled within the scope of this study. Roasted cocoa nibs were selected as the analytical matrix as they retain flavour-relevant precursors formed during fermentation and drying, while avoiding confounding effects introduced during chocolate manufacture. This study provides one of the first comprehensive compositional dataset for Australian cocoa nibs and offers new insights into producer-level variation in flavour-relevant chemistry within an emerging cocoa-producing region.

## 2 Materials and methods

### 2.1 Reagents and chemicals

All chemicals used in this study were of analytical reagent grade, and ultrapure water (18.2 MΩ·cm) was prepared on-site using a Milli-Q purification system (Millipore, North Ryde, NSW, Australia). High pressure liquid chromatography (HPLC)-grade solvents, methanol (>99.9%), sodium nitrite (NaNO_2_, ≥97%), aluminium chloride (AlCl_3_, 99%), ammonium ferrous sulfate ((NH₄)₂Fe(SO₄)₂·6H₂O, 99%), were obtained from Merck (Frenchs Forest, NSW, Australia). Formic acid (98%) was obtained from Rowe Scientific (Lonsdale, SA, Australia). Phenylacetaldehyde (≥97.5%, GC Grade) was obtained from Thermo Fisher Scientific (Illinois, US). All other reagents were obtained from Sigma-Aldrich (Darmstadt, Germany), unless otherwise stated, including acetic acid (glacial, >99.9%), 1,2-dichlorobenzene (99%), 2,3,5-trimethylpyrazine (99%), 2,3-butanedione (>99.9%), benzaldehyde (≥99.0%), 2-phenylethanol (≥99.0%), 2-methylpyrazine (≥99%), 2-furfural (≥98.5%), 5-methylfurfural (>99%), (−)-catechin (≥97%), protocatechuic acid (> 99%), vanillic acid (≥97%), vanillin, p-coumaric acid (≥98.0%), (−)-epicatechin (≥98%), α-ketoglutarate, theobromine (≥99.0%), pyruvic acid (98%), succinic acid (≥99%), 1,2‑dichlorobenzene‑*d*₄ (≥98%), lactic acid (≥98.0%), acetonitrile (≥99.9%), 2,2-diphenyl-1-picrylhydrazyl (DPPH, >90%), ethyl acetate (>99.8%), Folin-Ciocalteu reagent, gallic acid (>98%), hydrochloric acid (HCl, 37% *w*/w), iron (III) chloride (FeCl₃, >97%), propionic acid (>99.5%), sodium acetate (>99%), sodium carbonate (Na₂CO₃, >99.5%), sodium hydroxide (NaOH, >95%), sulfuric acid (H_2_SO_4_, 95.0%), 2,4,6-tripyridyl-*s*-triazine (TPTZ, >98%), and Trolox (>97%).

### 2.2 Sourcing cocoa nib samples

**Fig. 1:**
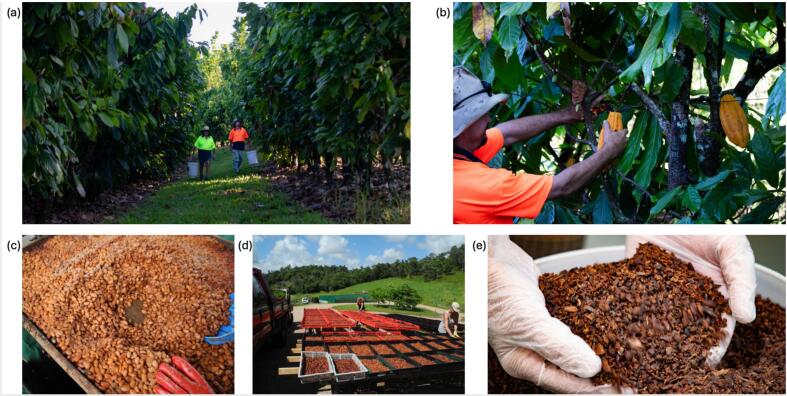
(a) Cocoa cultivation in Far North Queensland, Australia; (b) manual harvesting of mature cacao pods; (c) fermentation of freshly harvested cacao beans in wooden box fermenters; (d) sun-drying of fermented beans to reduce moisture prior to roasting; (e) roasted cocoa nibs used for chemical characterisation. Photo credit: Australian Chocolate Pty Ltd.

Dried and roasted cocoa nibs were produced by three commercial cocoa producers located in FNQ, Australia: Fishery Falls (FF), Shannonvale (SV), and Mount Edna (ME). The ME and SV plantations cultivate PNG hybrid cacao trees, whereas the FF plantation uses Queensland Department of Agriculture and Fisheries (QDAF) budwood grafted onto local rootstock (Fig. 1). All cocoa pods were harvested during the same summer season (2024–2025) to minimise seasonal variation. The ME, FF, and SV plantations comprise approximately 2000, 900, and 600 cocoa trees, respectively. The exact number of pods processed per commercial batch was not recorded. Freshly harvested cocoa pods from Fishery Falls were transported to the Mount Edna facility, where pods from both FF and ME were processed using the same fermentation, drying, and roasting facilities. Cocoa pods from SV were fermented, dried, and roasted at the SV production site. Representative cocoa nib samples were collected from each producer following commercial processing and therefore represent composite commercial production batches rather than individual fermentation batches. This study was designed to compare producer-level cocoa systems operating under broadly aligned commercial post-harvest practices, rather than to isolate genetic and environmental effects, reflecting commercial production practices in the region and the complexity of real-world cocoa production systems. Post-harvest processing was conducted using identical commercial processing protocols across all three origins. FF and ME were processed at the same facility, and SV was processed at the SV site using identical equipment and the same commercial protocol. However, fermentation and drying were conducted at two separate locations, and minor differences associated with commercial-scale operation, environmental conditions, and spontaneous microbial communities could not be controlled.

Fermentation was performed in square wooden box fermenters (900 × 400 × 600 mm) under ambient conditions without supplemental heating, for a duration of five days. The fermentation followed a typical two-stage progression consisting of an initial anaerobic phase (approx. 24 to 48 h), during which freshly extracted cocoa beans were placed in fermentation boxes, covered with hessian, and left undisturbed. During this period, liquid ferment exudate (“sweatings”) drained naturally from the fermenting mass. The fermentation then transitioned into an aerobic phase (approx. 72 to 96 h), during which beans were turned twice to introduce oxygen and promote uniform fermentation. Internal fermentation temperatures reached a maximum of 46.5 °C during the fermentation process. Fermentation pH, microbial community composition, and oxygen concentrations, and bean mass per fermentation box were not monitored during the commercial production.

Immediately following completion of fermentation (within one hour), beans were sun-dried at the respective fermentation locations. Beans from FF and ME were dried at ME, while beans from SV were dried at SV. Sun-drying was conducted for 7 days until a target moisture content of approximately 6–7% was achieved. All dried beans were subsequently roasted using an LPG-fuelled ROASTMAX roaster (20 kg batch capacity) following an identical staged roasting profile to a final temperature of 120 °C, followed by cooling to ambient temperature. Detailed roasting duration, ramp-rate, and airflow data were not recorded as part of the commercial production records. Roasted beans were cracked and winnowed to separate nibs from husk material. The resulting cocoa nibs were transported to The University of Queensland and stored at −20 °C until further preparation and chemical analysis.

### 2.3 Sample preparation

#### 2.3.1 Preparation of cocoa nib powder

For each producer, six independent subsamples (*n* = 6) of cocoa nibs were prepared for non-volatile analyses, and three independent subsamples of cocoa nibs were prepared for volatile analysis. Each subsample was frozen at −80 °C in stainless steel containers and finely ground to a homogeneous powder using a TissueLyser II (QIAGEN, Tokyo, Japan) operated at a frequency of 30 s^−1^ for 10 s. Ground samples were immediately transferred to airtight containers and were stored at −80 °C until further analysis. All analytical methods used for the analysis of the cocoa nibs were adapted from previously published and validated protocols, as referenced in the corresponding methodology sections.

#### 2.3.2 Extraction methods for non-volatile compound analysis

##### 2.3.2.1 Methanolic extraction

Methanolic extracts were prepared for the determination of total phenolic content (TPC), total flavonoid content (TFC), targeted phenolic compounds, and the antioxidant assays as described by Wang et al. (2026). Approximately 2.0 g of cocoa nib powder (*n* = 6, accurately weighed) was extracted with 20 mL of 70% (*v*/v) methanol in 50 mL centrifuge tubes. Samples were sonicated in a water bath (Soniclean 160TD, Mektronics, Australia) for 15 min while operated at 330 W, then centrifuged at 4000 rpm for 10 min. The temperature of the water bath was kept below 25 °C using ice-cooling. The supernatant was filtered through a 0.45 μm filter and stored at −20 °C until further analysis. Extractions were performed on six independently prepared cocoa nib subsamples from each producer (SV, FF, and ME), yielding six independent extraction replicates per origin (*n* = 6). These replicates represent independent sample preparations and extractions rather than repeated instrumental measurements.

The use of aqueous methanol combined with ultrasonic-assisted extraction is widely employed for the extraction of phenolic compounds from plant-derived matrices and has previously been applied to the determination of phenolics, flavonoids, and antioxidant activity in food systems (Aboshora et al., 2014; Altemimi et al., 2015; Kim & Lee, 2017; Luthria et al., 2007; Tahir et al., 2024; Wang et al., 2026). Ultrasonic-assisted extraction enhances mass transfer and cell disruption, thereby improving the recovery of phenolic compounds while reducing extraction time and solvent requirements (Tahir et al., 2024).

##### 2.3.2.2 Acidified aqueous extraction

Acidified aqueous extracts were prepared for organic acid analysis as described by Fernandez-Garcia and McGregor (1994) and Wang et al. (2026). Briefly, 50 mg (n = 6) of cocoa nib powder was extracted with 0.5 mL of 10 mM H_2_SO_4_ prepared in MilliQ water. Samples were briefly vortexed and then centrifuged at 15,000 rpm for 5 min. The supernatant was collected, and the extraction process was repeated twice on the remaining pellet. Combined supernatants were used for analysis.

### 2.4 Phenolic compounds

#### 2.4.1 Total phenolic content

The TPC was determined using the Folin-Ciocalteu reagent, as described by Ainsworth and Gillespie (2007). Methanol extracts (2.0 mL) or standard solutions (2.0 mL) were mixed with 0.2 N Folin-Ciocalteu reagent (0.4 mL) and incubated for 3 min at ambient temperature in the dark. Then, 7.5% (*w*/*v*) sodium carbonate was added, and the samples were incubated in the dark for 2 h. Absorbance was measured at 765 nm using a UV–vis spectrophotometer (Shimadzu UV–Vis 1800, Shimadzu, Kyoto, Japan). Gallic acid was used for calibration (0–250 μM). Calibration curves were converted to mass equivalents using molecular weight of gallic acid (170.12 g/mol), and the results were expressed as gallic acid equivalents per 100 g of dry weight cocoa nib powder (GAE/100 g DW).

#### 2.4.2 Total flavonoid content

The TFC were determined as described by Pękal and Pyrzynska (2014). Methanolic extracts (1.0 mL) or standard solutions (1 mL) were mixed with 0.3 mL 0.5% (w/v) NaNO_2_ and incubated for 5 min, followed by the addition of 0.5 mL 2% AlCl_3_ solution. After 6 min, 0.5 mL 1 M NaOH was added, and samples were incubated for a further 10 min. Absorbance was measured at 510 nm using a UV–Vis spectrophotometer (Shimadzu UV–Vis 1800, Shimadzu, Kyoto, Japan). Catechin was used as the reference standard (0–100 μg/mL) and the result expressed as catechin equivalents per 100 g of dry weight cocoa nib powder (mg CE/100 g DW).

#### 2.4.3 Targeted phenolic compounds

The target phenolic compounds were quantified using HPLC with diode-array detection (DAD) as described by Gasperotti et al. (2015) with slight modification. Briefly, the methanolic extracts were centrifuged at 16,000 x*g* for 5 min at 4 °C prior to analysis. Separation was achieved using a Waters Acquity HSS T3 column (100 × 2.1 mm, 1.8 μm). Mobile phase A consisted of 0.1% (*v*/v) formic acid in MilliQ water, and mobile phase B consisted of 0.1% formic acid in acetonitrile. The gradient program was as follows: 0 min, 5% B; 3 min, 20% B; 4 min, 20% B; 9 min, 45% B; 11 min, 100% B; 14 min, 100% B; 14.1 min, 5% B; and 20 min, 5% B. The flow rate was 0.4 mL/min, injection volume was 2 μL, and column temperature was maintained at 40 °C. Detection was carried out at 280, 320, and 360 nm. Quantification of protocatechuic acid, catechin, vanillic acid, vanillin, *p*-coumaric acid, gallic acid, epicatechin and theobromine was achieved using external calibration (0.7 μM - 3.0 mM). Data acquisition and integration were performed using Chromeleon software. Phenolic compounds are expressed as microgram per gram dry weight (μg/g DW).

### 2.5 Antioxidant capacity assays

Antioxidant capacity was assessed using the ferric reducing antioxidant power (FRAP) assay and the 2,2-diphenyl-1-picrylhydrazyl (DPPH) radical scavenging assay, using the 70% methanol extracts.

#### 2.5.1 Ferric reducing antioxidant power assay

The FRAP assay was conducted according to Benzie and Strain (1996) with minor modifications. The FRAP reagent was freshly prepared by mixing 300 mM acetate buffer (pH 3.6), 10 mM TPTZ in 40 mM HCl solution, and 20 mM FeCl_3_·6H_2_O in a 10:1:1 (v/v/v) ratio. Methanol extracts (1 mL) or standards (1 mL) were mixed with the FRAP reagent (2 mL) and incubated at 37 °C for 30 min. All standards and samples were analysed in duplicate. Absorbance was measured at 593 nm using a UV–Vis spectrophotometer (Shimadzu UV–Vis 1800, Shimadzu, Kyoto, Japan). Ammonium ferrous sulfate ((NH₄)₂Fe(SO₄)₂·6H₂O) was used to construct calibration curves (0–700 μM). The results were expressed as Fe^2+^ equivalents per gram of dry weight of cocoa nib powder (μmol Fe^2+^ eq/g DW).

#### 2.5.2 2,2-Diphenyl-1-picrylhydrazyl radical scavenging assay

The DPPH assay was performed as described by Molyneux (2004). Briefly, 1 mM of the DPPH stock solution was diluted 1:10 with methanol to give a 0.1 mM solution prior to use. Methanol extracts were mixed with the DPPH solution and incubated for 30 min at room temperature in the dark. All standards and samples were analysed in duplicate. Absorbance was measured at 517 nm using a UV–Vis spectrophotometer (Shimadzu UV-1800, Shimadzu, Kyoto, Japan). Trolox was used as the reference standard (0–80 μM). Results were expressed as Trolox equivalents per gram of dry weight cocoa nib powder (μmol TE/g DW).

### 2.6 Organic acids

The organic acids were quantified using the method described by Fernandez-Garcia and McGregor (1994), using a Thermo Vanquish Duo HPLC-DAD and absorbance was measured at 210 nm wavelength. Separation was achieved using an Agilent HiPlex H column (300 × 7.7 mm H^+^ form) with a Phenomenex Security Guard Carbo-H column. Isocratic elution was performed for 26 min using a mobile phase consisting of 4 mM H_2_SO_4_ at a flow rate of 0.6 mL/min. Organic acids, including acetic acid, formic acid, lactic acid, succinic acid, and pyruvic acid were identified and quantified using external calibration (0.15–100 mM). No internal standard was used. Data acquisition and integration were performed using Chromeleon software. Results were expressed as milligrams per gram of dry weight cocoa nib powder (mg/g DW).

### 2.7 Volatile aroma compounds analysis

#### 2.7.1 Untargeted semi-quantitative headspace solid phase microextraction gas-chromatography mass spectrometry

Volatile compounds were analysed using headspace solid-phase microextraction gas chromatography–mass spectrometry (HS-SPME-GC–MS) following a modified method of Ma et al. (2021). Similar HS-SPME-GC–MS approaches have been successfully applied for volatile profiling of fermented food and beverage matrices, demonstrating the suitability of this technique for comprehensive aroma characterisation (Roufa et al., 2026). Three independently prepared cocoa nib subsamples (*n* = 3) from each producer were analysed by HS-SPME-GC–MS were weighed (0.5 g) into 20 mL glass headspace vials and sealed with PTFE/silicone septa. Samples were stored at −80 °C prior to analysis to minimise volatile loss and compositional changes. Each subsample was extracted and analysed separately and does not represent repeated instrumental injections of the same vial. Prior to extraction, 1,2‑dichlorobenzene‑*d*₄ (5 μL, 100 μM in ethanol) was added to each vial as an internal standard. Samples were equilibrated at 60 °C for 10 min agitation, followed by headspace extraction for 15 min using an SPME Arrow fibre coated with 120 μm divinylbenzene/carboxen/polydimethylsiloxane (DVB/CAR/PDMS). Prior to use, the fibre was conditioned according to the manufacturer's recommendations. Thermal desorption of analytes was performed in the GC injector at 250 °C for 1 min in split mode (1:5). GC–MS analysis was conducted using a Shimadzu GCMS-TQ8050 NX system equipped with an SH-Rxi-5Sil MS capillary column. The oven temperature program was as follows: initial temperature 50 °C (5 min), increased to 250 °C at 10 °C min^−1^, and held for 5 min. Mass spectrometric detection was performed using electron ionisation at 70 eV. Data acquisition was conducted in multiple reaction monitoring (MRM) mode to enhance sensitivity and selectivity based on predefined transitions from the Shimadzu Smart Aroma Database. Compound identification was based on the comparison of mass spectra with the Flavour and Fragrance Natural and Synthetic Compounds Library (FFNSC 4, Shimadzu Corporation) and retention indices calculated under the analytical conditions employed (Table S1). Peak areas were normalised to the internal standard and expressed as relative abundance (%), enabling comparison of volatile profiles among cocoa samples.

#### 2.7.2 Targeted quantification of aroma-active compounds

Targeted volatile analysis was performed using the HS-SPME-GC–MS method described in Section 2.7.1 to quantify selected aroma-active compounds relevant to cocoa flavour, including 2,3-butanedione, ethyl acetate, 2-phenylethanol, phenylacetaldehyde, 2-methylpyrazine, furfural, benzaldehyde, and 2,3,5-trimethylpyrazine. Authentic analytical standards were used for compound identification and quantification. Stock solutions of each compound (10 mM) were prepared in ethanol and serially diluted to generate an eleven-point calibration range (0.0976–100 μM). Calibration standards (1 mL) were transferred into 20 mL sealed headspace vials and analysed under identical HS-SPME-GC–MS conditions as the cocoa samples. For sample analysis, ground cocoa nibs (0.5 g) were placed into 20 mL headspace vials and spiked with 1,2‑dichlorobenzene‑*d*₄ (5 μL of 100 μM in ethanol) as an internal standard prior to extraction. All samples and standards were equilibrated and extracted under identical conditions to ensure comparable headspace partitioning behaviour.

Quantification was performed using external calibration with internal standard normalisation. Volatile concentrations are reported as semi-quantitative headspace-equivalent estimates derived from solvent-based calibration. Volatile abundances were estimated using calibration curves (R^2^ > 0.99) and are reported as headspace-equivalent concentration estimates (μM) in sealed vials at equilibrium. Quantification by HS-SPME is governed by matrix-dependent partitioning equilibria, and the use of liquid calibration standards does not fully account for matrix effects, which may influence analyte recovery and fibre uptake (Davis & Qian, 2019). These effects are particularly relevant for solid food matrices, where sorption and diffusion processes further alter headspace composition (Higashikawa et al., 2013). Consequently, the reported headspace-equivalent concentration estimates should be interpreted as semi-quantitative measurements of abundance. Nevertheless, under controlled and consistent analytical conditions, HS-SPME provides reproducible and scientifically valid comparative data across samples

### 2.8 Data processing and statistical analysis

The non-volatile dataset comprised six independent extraction replicates per producer (*n* = 6), while volatile analyses comprised three independently prepared HS-SPME sample replicates per producer (*n* = 3). Replicates represent independent sample preparations and analyses rather than repeated instrumental measurements. All non-volatile and volatile analytical results were expressed as mean ± standard deviation. Statistical analyses were performed using GraphPad Prism v10.4.2 (GraphPad Software, Boston, MA, USA). One-way analysis of variance (ANOVA) followed by Tukey post-hoc testing was applied, with significance defined as (* *p* ≤ 0.05; ** *p* ≤ 0.01; *** *p* ≤ 0.001; **** *p* ≤ 0.0001).

Untargeted volatile data processing was performed using LabSolutions Insight (v4.0 SP6, Shimadzu, Kyoto, Japan). Peak areas were normalised to the internal standard to correct for analytical variability. The resulting semi-quantitative dataset was subjected to multivariate statistical analysis using MetaboAnalyst 5.0 (Chong et al., 2018). Prior to analysis, data were log_10_ transformed to stabilise variance and Pareto scaled to decrease the influence of large-magnitude variables while retaining data structure. Hierarchical clustering analysis (HCA), principal component analysis (PCA), and partial least squares discriminant analysis (PLS-DA) were performed to visualise differences in volatile profiles among cocoa nibs from the three production locations. PLS-DA model performance was evaluated using 5-fold cross-validation, and variable importance in projection (VIP) scores were calculated to identify compounds contributing most strongly to sample discrimination. Model robustness was evaluated using cross-validation, and variable importance in projection (VIP) scores were calculated to identify compounds contributing most strongly to sample discrimination. Heat maps were generated based on scaled data to visualise relative abundance patterns of volatile compounds across samples.

## 3 Results and discussion

The cocoa nibs investigated in this study were sourced from three commercial producers in FNQ and processed using a standardised post-harvest protocol. The beans from FF and ME were fermented and dried at the same facility, whereas the beans from SV were fermented at a different site using the same protocol. The ME and SV plantations cultivate PNG hybrid cacao trees, while FF utilises QDAF budwood grafted on local rootstock. As fermentation relies on spontaneous microbial activity, some degree of compositional variability was expected under these commercial processing conditions.

### 3.1 Phenolic compounds

#### 3.1.1 Total phenolic content and total flavonoid content

**Fig. 2:**
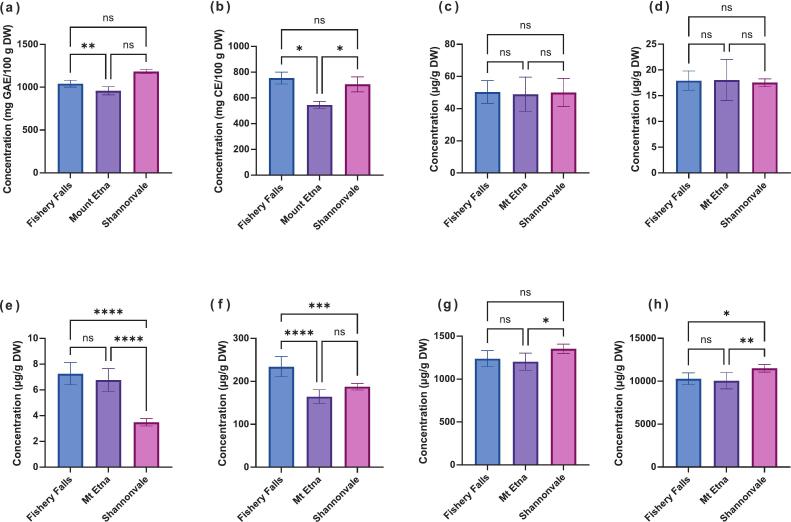
Total phenolic content (TPC), total flavonoid content (TFC), selected phenolic compounds, and methylxanthines in cocoa nibs from three Far North Queensland producers. (a) TPC, expressed as mg gallic acid equivalents per 100 g dry weight (mg GAE/100 g DW); (b) TFC, expressed as mg catechin equivalents per 100 g dry weight (mg CE/100 g DW); (c) protocatechuic acid (μg/g DW); (d) vanillin (μg/g DW); (e) *p*-coumaric acid (μg/g DW); (f) vanillic acid (μg/g DW); (g) (−)-epicatechin (μg/g DW); and (h) theobromine (μg/g DW). Values are presented as mean ± standard deviation (*n* = 6). Different letters indicate significant differences between producers (p < 0.05; one-way ANOVA with Tukey's post-hoc test).

Producer-dependent TPC and TFC differences were observed in the cocoa nibs obtained from three FNQ producers (Fig. 2a, b). Mount Edna (ME) exhibited significantly lower TPC and TFC than Fishery Falls (FF) and Shannonvale (SV), while no differences were observed between FF and SV. Phenolic compounds are key compositional markers in cocoa, with reported TPC values for cocoa nibs typically ranging from ∼1000 to 3000 mg GAE per 100 g dry weight depending on genotype and post-harvest processing (Siow et al., 2022; Souza et al., 2024). The values obtained in this study (957.84–1184.12 mg GAE/100 g DW) fall within the lower to mid-range of these reported values. Similarly, TFC values were lower in ME (272.39 mg CE/100 g DW) compared with FF and SV (∼352–370 mg CE/100 g DW), consistent with the expected transformation of flavan-3-ols during fermentation and drying (Siow et al., 2022; Souza et al., 2024). Catechin was used as the calibration standard to reflect the dominance of flavan-3-ols in cocoa. The decreased TPC and TFC observed in ME likely reflect increased oxidative transformation of phenolic compounds during fermentation, where enzymatic oxidation (e.g., polyphenol oxidase activity) promotes quinone formation and subsequent polymerisation or binding to macromolecules, reducing extractable phenolics. In contrast, higher values in FF and SV indicate greater retention of extractable phenolic material. Despite differences in genetic background and fermentation location, FF and SV exhibited comparable phenolic contents, suggesting that phenolic retention is governed by the combined influence of raw material characteristics and site-specific fermentation dynamics rather than a single dominant factor. The parallel trends observed for TPC and TFC further indicate that variation is primarily driven by changes in the flavonoid-rich fraction, consistent with the known susceptibility of flavan-3-ols to oxidative degradation during cocoa processing (Hurst et al., 2011).

The producer-level differences observed in TPC and TFC indicate that Australian cocoa nibs are not chemically uniform despite originating from the same broad production region. While the variation was modest, the lower phenolic and flavonoid contents observed in ME relative to FF and SV suggest that local production and post-harvest conditions may influence phenolic retention. Similar processing-related effects on phenolic composition and antioxidant activity have been reported in cocoa from other producing regions, where factors such as depulping and roasting influence the retention of bioactive compounds (Asiedu et al., 2025). Importantly, all values remained within internationally reported ranges, indicating that Australian cocoa possesses a relatively consistent compositional baseline while retaining measurable producer-level variation that may support future provenance and traceability applications.

#### 3.1.2 Targeted phenolic compounds and methylxanthines

Targeted analysis of eight phenolic and methylxanthine compounds identified six consistently across all samples: protocatechuic acid, vanillic acid, vanillin, *p*-coumaric acid, epicatechin, and theobromine (Fig. 2, Table 1).

**Table 1:** Concentrations of chemical composition in Australian cocoa nibs.

	Fishery Falls	Mount Edna	Shannonvale
Total phenolic content (mg GAE/100 g DW)	1042.16 ± 39.68 ^b^	957.84 ± 47.82 ^b^	1184.12 ± 22.96 ^a^
Total flavonoid content (mg CE/100 g DW)	369.75 ± 27.97 ^a^	272.39 ± 12.91 ^b^	352.447 ± 25.35 ^a^
DPPH Antioxidant assay (μmol TE/g DW)	849.97 ± 90.50 ^b^	710.52 ± 43.20 ^c^	964.48 ± 56.20 ^a^
FRAP Antioxidant assay (μmol Fe^+2^ eq/g DW)	23.66 ± 0.52 ^b^	21.03 ± 1.56 ^c^	29.51 ± 0.45 ^a^
Protocatechuic acid (μg/g DW)	50.37 ± 7.06 ^a^	48.92 ± 10.69 ^a^	50.03 ± 8.81 ^a^
Vanillin (μg/g DW)	17.94 ± 1.87 ^a^	18.06 ± 4.02 ^a^	17.55 ± 0.74 ^a^
Vanillic acid (μg/g DW)	234.14 ± 23.40 ^a^	164.33 ± 16.03 ^b^	187.71 ± 7.76 ^b^
*p*-Coumaric acid (μg/g DW)	7.26 ± 0.86 ^a^	6.77 ± 0.88 ^a^	3.48 ± 0.29 ^b^
Epicatechin (μg/g DW)	1238.31 ± 93.49 ^ab^	1202.83 ± 99.56 ^b^	1353.02 ± 53.65 ^a^
Theobromine (μg/g DW)	10,292.91 ± 676.11 ^b^	10,062.18 ± 953.52 ^b^	11,514.61 ± 458.35 ^a^
Acetic acid (mg/g DW)	2.65 ± 0.95 ^b^	3.04 ± 0.087 ^b^	5.16 ± 0.64^a^
Formic acid (mg/g DW)	0.11 ± 0.008 ^a^	0.11 ± 0.004 ^a^	0.10 ± 0.005 ^a^
Lactic acid (mg/g DW)	1.31 ± 0.13 ^a^	1.29 ± 0.22 ^a^	0.52 ± 0.24 ^b^
Succinic acid (mg/g DW)	0.24 ± 0.012 ^b^	0.25 ± 0.054 ^b^	0.36 ± 0.043 ^a^
Pyruvic acid (mg/g DW)	0.044 ± 0.023 ^a^	0.053 ± 0.018 ^a^	0.054 ± 0.018 ^a^
2,3-Butanedione (μM)⁎	35.68 ± 0.52 ^b^	53.23 ± 0.89 ^a^	29.59 ± 1.45 ^c^
Ethyl acetate (μM)⁎	51.67 ± 1.40 ^c^	80.83 ± 8.23 ^b^	176.07 ± 6.00 ^a^
2-Phenylethanol (μM)⁎	77.50 ± 1.70 ^a^	52.40 ± 11.70 ^b^	46.43 ± 2.77 ^b^
2-Methylpyrazine (μM)⁎	0.31 ± 0.01 ^b^	0.31 ± 0.01 ^b^	0.45 ± 0.01 ^a^
2,3,5-Trimethylpyrazine (μM)⁎	2.47 ± 0.05 ^a^	3.46 ± 0.20 ^a^	2.72 ± 0.07 ^b^
Furfural (μM)⁎	0.61 ± 0.01 ^b^	0.61 ± 0.01 ^b^	0.91 ± 0.10 ^a^
Benzaldehyde (μM)⁎	1.70 ± 0.11 ^a^	1.91 ± 0.11 ^a^	0.98 ± 0.03 ^b^
Phenylacetaldehyde (μM)⁎	0.68 ± 0.02 ^ab^	0.66 ± 0.06 ^b^	0.77 ± 0.03 ^a^

Values are expressed as mean ± standard deviation (*n* = 6 for non-volatile analysis, and n = 3 for HS-SPME-GC–MS analysis as described in Section 2.3.1, Section 2.7, and Section 2.8). Different superscript letters within a row indicate significant differences between origins (p < 0.05).

⁎ Volatile compounds are reported as headspace-equivalent concentration estimates (μM) measured in sealed 20 mL vials under the HS-SPME-GC–MS conditions described in Section 2.7. Volatile concentrations are reported as semi-quantitative headspace-equivalent estimates derived from solvent-based calibration. Values should be interpreted as comparative indicators of volatile abundance rather than absolute concentrations in the cocoa matrix.

The low-molecular-weight phenolic acids were present at comparable concentrations across origins. Protocatechuic acid (∼48.9–50.4 μg/g DW, Fig. 2c, Table 1) and vanillin (∼17.5–18.1 μg/g DW, Fig. 2d, Table 1) did not differ significantly between producers (*p* > 0.05), indicating a high degree of compositional consistency for these degradation-derived phenolics. These compounds are recognised as secondary products of flavan-3-ol oxidation and lignin-derived phenolic metabolism during fermentation and drying, and are typically observed at low μg/g levels in cocoa (Jalil & Ismail, 2008; Afoakwa et al., 2008). The concentrations measured here align with values reported for fermented and roasted cocoa from major producing regions (Souza et al., 2024).

In contrast, selective origin-dependent differences were observed for specific phenolic acids. *p*-Coumaric acid was significantly lower in SV (3.48 ± 0.29 μg/g DW) compared with ME (6.77 ± 0.88 μg/g DW) and FF (7.26 ± 0.86 μg/g DW) (*p* < 0.05, Fig. 2e, Table 1). Similarly, vanillic acid ranged from 164.33 ± 16.03 μg/g DW (ME) to 234.14 ± 23.40 μg/g DW (FF), with FF significantly higher than the other producers (Fig. 2f, Table 1). These differences likely variation in precursor availability and phenolic transformation during fermentation, as it is known that hydroxycinnamic acids are sensitive to enzymatic oxidation and microbial metabolism (Afoakwa et al., 2008; Jalil & Ismail, 2008). However, the relative contribution of enzymatic oxidation and microbial metabolism cannot be resolved here. Epicatechin, the dominant flavan-3-ol in cocoa, was the most abundant quantified phenolic compound, with concentrations ranging from 1202.83 ± 99.56 to 1353.02 ± 53.65 μg/g DW (Fig. 2g, Table 1). SV exhibited significantly higher levels than ME (p < 0.05), while FF showed intermediate values. This trend is consistent with the known sensitivity of epicatechin to fermentation and oxidation, where partial degradation and polymerisation to procyanidins can occur (Afoakwa et al., 2008; Hermund et al., 2025). The concentrations reported here are comparable to those observed in roasted cocoa nibs by Souza et al. (2024), supporting the compositional equivalence of Australian-grown cocoa to established international origins.

The methylxanthine, theobromine, was the most abundant compound overall, with concentrations of approximately 10,062–10,293 μg/g DW (Fig. 2h, Table 1). SV exhibited significantly higher theobromine content compared with ME and FF (*p* < 0.05), which did not differ from each other. As a methylxanthine synthesised during bean development, theobromine is largely stable during fermentation and roasting, and its concentration is primarily governed by genetic factors, with limited modification during post-harvest processing (Afoakwa et al., 2008; Hermund et al., 2025). Comparable concentration ranges and origin-dependent variation have been reported for cocoa from South America and Southeast Asia (Souza et al., 2024). Given its recognised role as bitter tasting methylxanthine (Stark et al., 2006), the elevated theobromine levels observed in Shannonvale may contribute to differences sensory perception, particularly when considered alongside organic acid composition.

The differences observed in vanillic acid, *p*-coumaric acid, epicatechin, and theobromine suggest that targeted phenolic and methylxanthine profiling may provide greater discriminatory power than bulk TPC and TFC measurements. While all concentrations were within internationally reported ranges, the producer-specific patterns observed here indicate that individual metabolites may contribute to future provenance and authentication frameworks for Australian-grown cocoa. Similar approaches have been proposed for quality differentiation and traceability of premium food products, where targeted compositional markers often provide greater resolution than bulk compositional indices (Asiedu, Tutu, Afoakwa, Akonor, Budu, & Saalia, 2026; Asiedu, Tutu, Afoakwa, Akonor, Owusu-Bempah, et al., 2026). Further studies incorporating additional producers, production batches, and harvest seasons will be required to assess the robustness of these compounds as origin markers.

#### 3.1.3 Antioxidant capacity

**Fig. 3:**
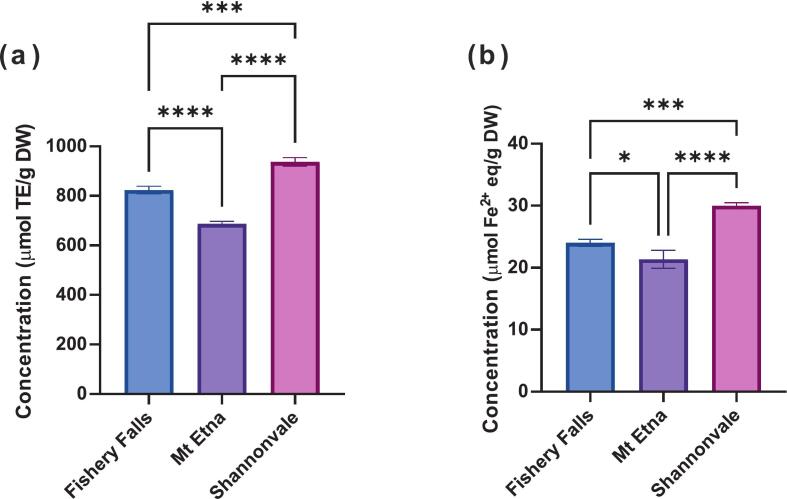
(a) Radical scavenging activity determined using the DPPH assay, expressed as μmol Trolox equivalents per g dry weight (μmol TE/g DW), and (b) the ferric reducing antioxidant power (FRAP), expressed as μmol Fe^2+^ eq per g dry weight (μmol Fe^2+^/g DW). Values are presented as mean ± standard deviation (*n* = 6). Different letters indicate significant differences between producers (*p* < 0.05; one-way ANOVA with Tukey's post-hoc test).

Significant differences in the antioxidant capacity were observed in the cocoa nibs from the three producers, as determined by both DPPH and FRAP assays (Fig. 3, Table 1). The nibs from SV consistently exhibited the highest antioxidant capacity across both assays (DPPH: 964.48 ± 56.20 μmol TE/g DW; FRAP: 29.51 ± 0.45 μmol Fe^2+^/g DW, Fig. 3, Table 1), followed by FF, while ME showed significantly lower values (*p* < 0.05). This trend aligns with compositional data, where SV also displayed the highest total phenolic content and elevated epicatechin levels, supporting the central role of flavan-3-ols in determining antioxidant capacity in cocoa.

Although both assays showed consistent ranking across producers, differences in magnitude reflect their distinct mechanisms. The DPPH assay primarily measures radical scavenging by low-molecular-weight phenolics, whereas FRAP reflects the cumulative reducing capacity of a broader pool of redox-active compounds. Such differences are expected and provide complementary insight into antioxidant behaviour.

The antioxidant capacities measured in this study are broadly consistent with reported ranges in the literature, although absolute values differ substantially due to methodological differences in extraction, calibration, and reporting units. Souza et al. (2024) reported DPPH values of approximately 400–600 μmol TE/g and FRAP values of 160–290 μmol/g, while Siow et al. (2022) demonstrated substantial variability associated with origin and roasting conditions. The slightly higher DPPH values observed here likely reflect differences in extraction efficiency and phenolic composition. Earlier studies (e.g., Jonfia-Essien et al., 2008) report lower FRAP values; however, these differences are attributable to variation in extraction protocols, calibration standards, and sample processing state, and are therefore not directly comparable.

Overall, these results confirm that Australian cocoa nibs exhibit antioxidant capacities comparable to established cocoa-producing regions, while also showing meaningful producer-level variation. The higher antioxidant capacity observed in SV is consistent with its higher TPC and epicatechin content, supporting the role of flavan-3-ols as major contributors to antioxidant behaviour in cocoa. Similar relationships between processing, phenolic composition, and antioxidant activity have been reported in cocoa from other producing regions (Asiedu et al., 2025). Together, these findings suggest that antioxidant measurements may provide useful complementary markers for producer-level quality characterisation when interpreted alongside targeted phenolic profiles.

### 3.2 Organic acids

**Fig. 4:**
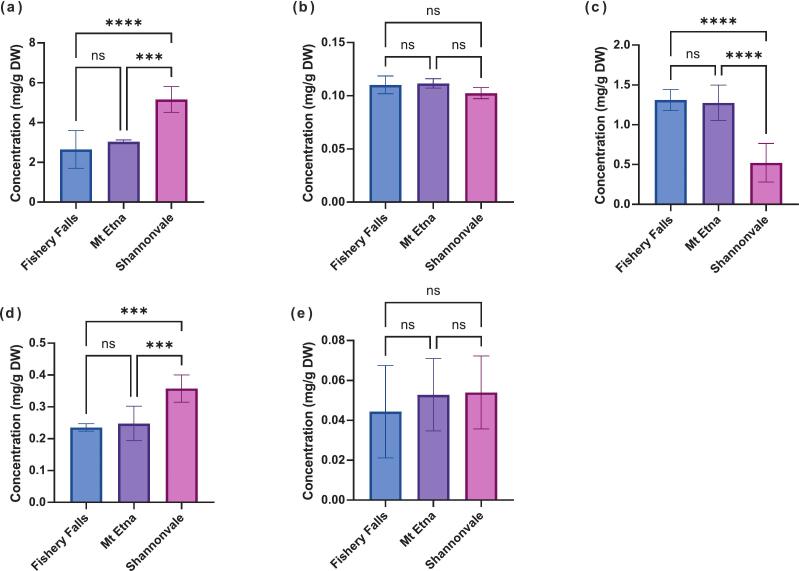
Organic acid profiles of cocoa nibs from three Far North Queensland producers (Fishery Falls, Mount Edna, and Shannonvale): (a) acetic acid (mg/g DW), (b) formic acid (mg/g DW), (c) lactic acid (mg/g DW), (d) succinic acid (mg/g DW), and (e) pyruvic acid (mg/g DW). Values are presented as mean ± standard deviation (n = 6). Different letters indicate significant differences between producers (p < 0.05; one-way ANOVA with Tukey's post-hoc test).

Acetic and lactic acids were the dominant organic acids across all cocoa nib samples, with succinic acid present at intermediate levels and pyruvic and formic acids at low concentrations (Fig. 4, Table 1). Significant producer-level differences were observed for acetic, lactic, and succinic acids, while pyruvic and formic acids did not differ (*p* > 0.05). FF and ME exhibited similar profiles characterised by higher lactic acid (1.31 ± 0.13 and 1.29 ± 0.22 mg/g DW, Fig. 4c, Table 1) and lower acetic acid (2.65 ± 0.95 and 3.04 ± 0.09 mg/g DW, Fig. 4a, Table 1). In contrast, Shannonvale (SV) showed significantly lower lactic acid (0.52 ± 0.24 mg/g DW, Fig. 4c, Table 1) and higher acetic acid (5.16 ± 0.64 mg/g DW) (Fig. 4a, Table 1), indicating a pronounced shift in the lactic-to-acetic acid balance. As FF and ME were fermented at the same facility, while SV was processed at a different site, these differences suggest site-specific effects on fermentation outcomes. Lactic acid is produced during early anaerobic stages by lactic acid bacteria, whereas acetic acid arises during later aerobic phases via ethanol oxidation by acetic acid bacteria (De Vuyst & Leroy, 2020; Ho et al., 2014). Increased acetic acid therefore indicates greater oxidative conditions during the aerobic fermentation phase. From a quality perspective, controlled acetic acid formation is important for flavour precursor development. Previous studies have associated elevated acetic acid concentrations with increased acidity and higher lactic acid concentrations with milder sensory profiles (Afoakwa et al., 2008). Similar fermentation-dependent changes in organic acid composition have been reported for CCN51 cocoa beans, where fermentation and drying practices significantly influenced acid accumulation and flavour development (Flores et al., 2025).Succinic acid was higher in SV (0.36 ± 0.04 mg/g DW) than in FF and ME (∼0.24–0.25 mg/g DW) (Fig. 4d, Table 1), consistent with enhanced microbial activity. Formic and pyruvic acids remained low and unchanged across producers (Fig. 4b, e, Table 1).

Overall, organic acid concentrations were within ranges reported for fermented cocoa (Mohamed et al., 2019; Roda & Lambri, 2019; Velásquez-Reyes et al., 2023). However, the distinct lactic-to-acetic acid ratios observed among producer lots indicate measurable differences in fermentation outcomes despite broadly aligned post-harvest practices. Similar studies have highlighted the importance of processing-derived compositional markers for product differentiation and quality characterisation (Asiedu, Tutu, Afoakwa, Akonor, Budu, & Saalia, 2026). Accordingly, organic acid profiles may provide useful indicators of producer-level variation and future provenance characterisation of Australian-grown cocoa.

### 3.3 Volatile organic compound analysis

#### 3.3.1 Targeted quantification of key aroma-active compounds

**Fig. 5:**
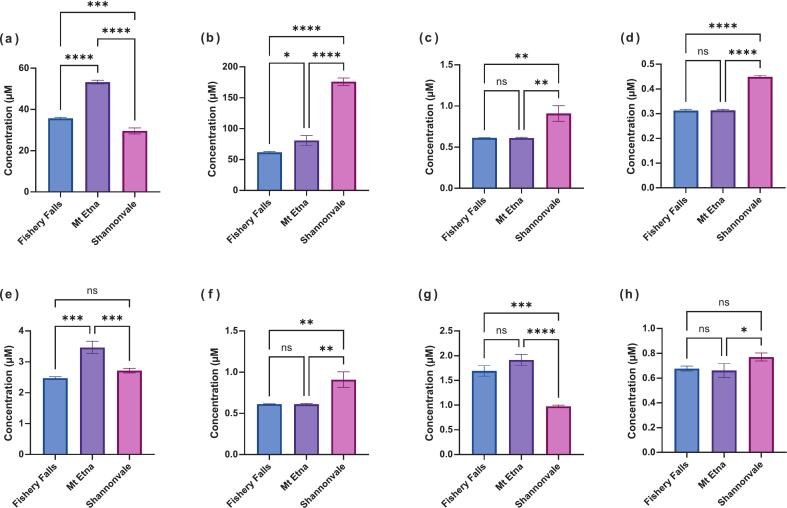
Headspace-equivalent concentration estimates (μM) of selected volatile compounds in cocoa nibs from three Far North Queensland producers (Fishery Falls, Mount Edna, and Shannonvale): (a) 2,3-butanedione, (b) ethyl acetate, (c) 2-phenylethanol, (d) 2-methylpyrazine, (e) 2,3,5-trimethylpyrazine, (f) furfural, (g) benzaldehyde, and (h) phenylacetaldehyde. Values are presented as mean ± standard deviation (*n* = 3). Different letters indicate significant differences between producers (*p* < 0.05; one-way ANOVA with Tukey's post-hoc test).

Targeted quantification of key aroma-active volatile compounds, namely 2,3-butanedione, ethyl acetate, 2-phenylethanol, 2-methylpyrazine, 2,3,5-trimethylpyrazine, furfural, benzaldehyde, and phenylacetaldehyde, revealed clear and origin-dependent differences among cocoa nibs from FF, ME, and SV, likely reflecting variation in fermentation- and processing-derived chemistry (Fig. 5, Table 1). The selected aroma-active compounds are widely reported as aroma-active constituents of cocoa and are frequently used as markers of fermentation- and roasting-related chemistry (Afoakwa et al., 2008). These include fermentation-derived alcohols, esters, and diketones, as well as roasting-derived pyrazines and furan compounds (Afoakwa et al., 2008).

Among fermentation-associated volatiles, significant differences were observed for 2,3-butanedione, ethyl acetate, and 2-phenylethanol (Table 1). Relative abundance of 2,3-butanedione were significantly higher in ME compared with FF and SV (Fig. 5a, Table 1), consistent with greater contributions from lactic acid bacteria (LAB)-associated metabolic pathways. It is important to note that microbial populations were not directly characterised in this study, and therefore mechanistic interpretations are inferred from established fermentation pathways. 2,3-Butanedione is formed via the pyruvate–α-acetolactate pathway during LAB activity and is typically associated with lactic fermentation pathways under moderate oxygen availability (Dulce et al., 2021; Ho et al., 2014). This compound has previously been associated with buttery or creamy aroma notes in cocoa and related matrices (Afoakwa et al., 2008). Its elevated levels in ME are consistent with the higher lactic acid concentrations measured in these nibs, supporting a comparatively balanced and comparatively less oxidative fermentation regime. Ethyl acetate was present in significantly higher concentrations in SV compared with ME and FF (Fig. 5b, Table 1), indicating enhanced yeast-driven ester formation. Ethyl acetate is mainly formed during early anaerobic fermentation through the conversion of ethanol, although secondary formation via esterification involving acetic acid bacteria may also occur during later stages (De Vuyst & Leroy, 2020;Ho et al., 2014; Mendoza Salazar et al., 2022). Ethyl acetate has previously been associated with fruity and solvent-like notes, depending on concentration (Afoakwa et al., 2008). Its elevated levels in SV align with the higher acetic acid concentrations measured in these nibs, suggesting differences in fermentation dynamics, including potential variation in microbial metabolism and ethanol oxidation pathways. In contrast, 2-phenylethanol was significantly higher in FF compared with ME and SV (Fig. 5c, Table 1). This compound is produced by yeasts via the Ehrlich pathway from *l*-phenylalanine and is widely recognised as a marker of yeast-derived aroma development in cocoa fermentation (Ho et al., 2014; Mendoza Salazar et al., 2022). It has been reported to contribute floral and rose-like aroma characteristics in cocoa and other fermented products (Afoakwa et al., 2008). Its higher abundance in FF suggests more sustained or efficient yeast metabolism during the early stages of fermentation.

Roasting-derived compounds also exhibited clear origin-dependent differences. Pyrazines, formed through Maillard reactions between amino acids and reducing sugars during drying and roasting, varied significantly among samples. 2-Methylpyrazine was significantly higher in SV, while 2,3,5-trimethylpyrazine was highest in ME (Fig. 5d, e, Table 1). Pyrazines are widely recognised as aroma-active compounds associated with roasted, nutty, and cocoa-like aromas and these differences likely reflect variation in precursor availability established during fermentation, which governs subsequent Maillard chemistry (Afoakwa et al., 2008;Jinap et al., 1995; Stark et al., 2006).

Furan compounds and Strecker-derived aldehydes further supported these trends. Furfural was significantly higher in SV, whereas benzaldehyde was highest in ME and lowest in SV (Fig. 5f, g, Table 1). Furfural has been associated with sweet, caramel-like notes, while benzaldehyde has been associated with contributes almond-like aromas (Stark et al., 2006). Furan compounds and Strecker-derived compounds arise from thermal degradation of sugars and amino acids, with fermentation influencing their formation indirectly through modulation of precursor pools (Jinap et al., 1995; Stark et al., 2006). In contrast, phenylacetaldehyde was significantly higher in Shannonvale than in Mount Edna, with Fishery Falls intermediate (Fig. 5h, Table 1). As a honey-like, floral Strecker aldehyde, this suggests modest origin-dependent variation in phenylalanine-derived aroma formation.

Collectively, these results suggest that, despite broadly standardised post-harvest processing, cocoa nibs from different Australian origins exhibit distinct volatile profiles. These results are consistent with differences in microbial activity during fermentation, which modulates precursor availability and ultimately governs the formation of key aroma-active compounds during roasting. The concentrations of volatile compounds reported here are consistent with the expected order of magnitude for fermented and roasted cocoa described in the literature (Afoakwa et al., 2008; Jinap et al., 1995; Stark et al., 2006), although direct comparison of absolute values is constrained by differences in analytical approaches, reporting units, and matrix–headspace partitioning behaviour. Accordingly, the values presented here should be interpreted as semi-quantitative headspace-equivalent concentration estimates reflecting relative volatile abundance and partitioning behaviour under the analytical conditions used, rather than absolute concentrations in the cocoa matrix. Interpretations regarding flavour relevance are based on aroma associations reported in the literature. As sensory evaluation was beyond the scope of the present study, differences in volatile composition should not be interpreted as evidence of perceptible sensory differences among cocoa nib samples.

#### 3.3.2 Untargeted semi-quantitative headspace analysis of the volatile compounds

**Fig. 6:**
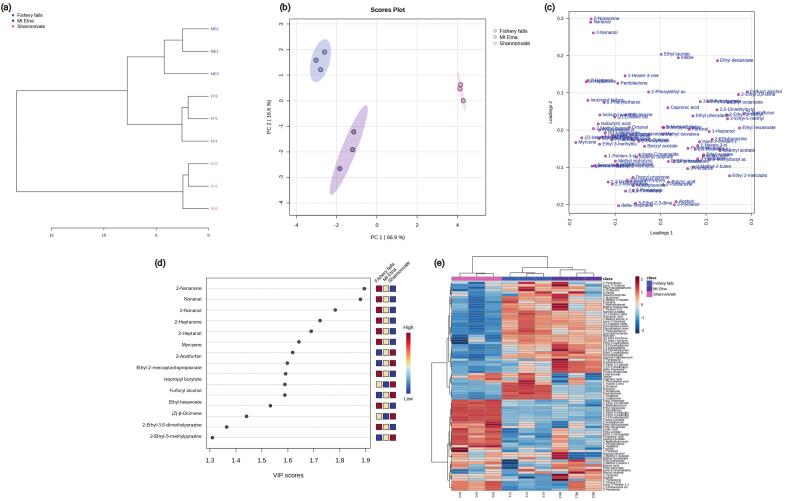
Multivariate analysis of untargeted volatile profiles obtained by HS-SPME-GC–MS from cocoa nibs produced by three Far North Queensland producers (Fishery Falls, Mount Edna, and Shannonvale). (a) Hierarchical clustering analysis (HCA) dendrogram showing similarity relationships among cocoa nib samples based on volatile composition. (b) Principal component analysis (PCA) scores plot illustrating separation of samples according to production location. (c) PCA loadings plot showing the volatile compounds contributing to the separation observed in the scores plot. (d) Variable importance in projection (VIP) scores derived from the exploratory PLS-DA model showing volatile compounds associated with producer-level discrimination. (e) Heat map with hierarchical clustering illustrating relative abundance patterns of volatile compounds across samples.

Untargeted HS-SPME-GC–MS analysis combined with multivariate statistics was used to characterise the volatile composition and to evaluate the differences between the nibs produced at the three locations (ME, FF, and SV) in FNQ, Australia. Hierarchical clustering analysis (HCA) showed separation among cocoa nibs from FF, ME, and SV (Fig. 6). HCA showed within-producer clustering, indicating good analytical reproducibility, and revealed two major groupings: FF and ME clustered together, while SV formed a distinct branch (Fig. 6a). This pattern indicates a higher similarity in overall volatile composition between FF and ME compared with SV, despite all samples undergoing comparable post-harvest processing.

The PCA scores further indicated producer-level differentiation in volatile composition. The first two principal components explained 83.5% of the total variance (PC1 66.6%, PC2 16.9%), separating the cocoa nib producers according to production location (Fig. 6b). The SV samples were distinctly separated from the other producers along PC1, while FF and ME grouped closer together but remained distinguishable from one another. The corresponding loadings shows that separation was associated with variation in the relative abundance of aldehydes, ketones, esters, pyrazines, and terpenes, reflecting differences in the relative contribution of fermentation-derived and thermally generated volatiles across producers.

Supervised multivariate analysis using PLS-DA was performed as an exploratory assessment of producer-specific differences. A two-component model selected by 5-fold cross-validation showed high explained variance (R^2^ = 0.997) and predictive ability (Q^2^ = 0.979), although model validation should be interpreted with caution due to the limited sample size. Consequently, the PLS-DA results are presented primarily as an exploratory visualisation of group differences rather than evidence of predictive classification performance. Variable importance in projection (VIP) analysis was subsequently used to identify the volatile compounds contributing most strongly to producer-level discrimination (Fig. 6c). These included roasting-associated compounds such as 2-ethyl-3,6-dimethylpyrazine, 2-ethyl-5-methylpyrazine, furfuryl alcohol, and 2-acetylfuran, which are typical Maillard-derived aroma compounds previously associated with roasted and nutty aroma characteristics in cocoa. Flores et al. (2025), for example, demonstrated that fermentation and drying conditions significantly influence the formation of key aroma-active compounds, such as pyrazines, esters, and aldehydes in cocoa beans. Lipid oxidation and fermentation-related compounds such as nonanal, 2-heptanal, 2-heptanone, 2-nonanone, and 2-nonanol also showed strong discriminatory power. In addition, fermentation-derived esters and alcohols including ethyl hexanoate and isopropyl butyrate contributed to sample differentiation, while terpene compounds such as (*Z*)-β-ocimene and myrcene likely reflect varietal or pod-derived aromatic contributions (Akoa et al., 2023; Quelal et al., 2023). Together, these compounds represent key markers associated with roasting chemistry, lipid-derived volatiles, fermentation metabolism, and inherent cocoa aromatic precursors.

A heat map based on Pareto-scaled volatile intensities further illustrated producer-specific differences in compound abundance (Fig. 6d). The clustering pattern was consistent with both HCA and PCA, with SV samples forming a distinct group, while FF and ME exhibited partial similarity. Several pyrazines, aldehydes, and fermentation-derived esters showed elevated relative abundance in specific producers, showing compound-level differences underlying the multivariate separation.

Notably, FF and ME samples clustered more closely together despite originating from different cacao genotypes, whereas SV and ME, which share the same PNG hybrid genotype, were separated in multivariate space. This pattern suggests that factors associated with the processing environment may have contributed to the observed producer-level variation. However, because genotype, fermentation conditions, environmental factors, and uncharacterised microbial communities were not independently controlled, these factors cannot be conclusively determined. The observed differences should therefore be interpreted as producer-level variation rather than evidence of specific causal mechanisms. In contrast, the closer similarity between FF and ME is consistent with the possibility that shared fermentation and drying environments contributed to similarities in volatile composition, however, this interpretation should be considered exploratory given the observational nature of this study. Together, these results demonstrate producer-level differences in volatile composition among the sampled cocoa nibs, which were associated with a consistent set of chemically meaningful volatile compounds.

A limitation of the present study is that sensory evaluation was not conducted. Consequently, interpretations regarding flavour relevance are based on established literature describing the sensory associations of individual compounds. While differences in volatile and non-volatile composition were observed among producers, the extent to which these differences translate into perceptible sensory differences remains to be determined through future sensory investigation. Furthermore, as samples were obtained from commercial producers, replication was based on independently prepared subsamples rather than multiple independently fermented production batches. Consequently, the results provide insight into producer-level compositional variation but do not permit statistical separation of within-producer and between-batch variability.

## 4 Conclusion

This study provides a comprehensive compositional characterisation of cocoa nibs produced in Far North Queensland, Australia, including phenolic compounds, methylxanthines, organic acids, antioxidant capacity, and volatile profiles. Across all compound classes, values were consistent with those reported for established cocoa-producing regions, supporting the compositional comparability of Australian-grown cocoa. Despite broadly standardised post-harvest processing, significant producer-level differences were observed, particularly in organic acids and volatile compounds, indicating that fermentation environment may contribute to compositional differentiation. However, it is important to note that while post-harvest processing conditions were broadly aligned, variation in genotype, drying location, and uncharacterised microbial communities associated with spontaneous fermentation represent potential sources of variability that could not be experimentally disentangled within the scope of this study. Multivariate analysis further suggests that samples were clustered according to fermentation location rather than genotype under the conditions studied, suggesting that fermentation context may contribute to in shaping flavour-relevant chemical profiles. Volatile concentrations reported here represent headspace-derived values and should be interpreted as indicators of relative abundance rather than absolute matrix concentrations. These findings establish a foundational compositional dataset for Australian cocoa and support the development of provenance-based differentiation within emerging cocoa industries. The findings also contribute to the broader understanding of quality differentiation in emerging cocoa-producing regions. As global cocoa production faces increasing supply challenges, such information may assist the development and evaluation of alternative cocoa-growing regions.

## Declaration of generative AI and language assistance

During the preparation of this manuscript, the authors used Grammarly (Grammarly Inc., San Francisco, CA, USA) to assist with grammar, spelling, and improvements in English language clarity. The authors subsequently reviewed and edited the manuscript in full and takes complete responsibility for the accuracy, integrity, and scientific content of the work.

## CRediT authorship contribution statement

**Jia Wang:** Writing – review & editing, Writing – original draft, Investigation, Formal analysis. **Marlize Zaretha Bekker:** Writing – review & editing, Writing – original draft, Visualization, Validation, Supervision, Resources, Project administration, Methodology, Investigation, Funding acquisition, Formal analysis, Data curation, Conceptualization.

## Funding

Funding for this research was provided by the 10.13039/501100001794University of Queensland (UQ) Building Industry Research Relationships in Science and Translation (BIRRST) grant.

## Declaration of competing interest

The authors declare that they have no known competing financial interests or personal relationships that could have appeared to influence the work reported in this paper.

## Data Availability

Data will be made available on request.
