# Supplementary material for: Compositional profiling of volatile and non-volatile compounds in Australian cocoa nibs: Insights into origin-dependent variability
Source: Food Chem X. 2026 Jul 16;38:104220. doi: 10.1016/j.fochx.2026.104220 (PMC13393560; doi:10.1016/j.fochx.2026.104220)
Supplement: Supplementary file 1 — Supplementary material. [file mmc1.docx]

**Supporting information for**

**Compositional profiling of volatile and non-volatile compounds in Australian cocoa nibs: insights into producer-level variability**

Jia Wang^1^ and Marlize Zaretha Bekker^1*^

^1^ School of Agriculture and Food Sustainability, The University of Queensland, St Lucia, QLD 4072, Australia

*Corresponding author: Dr Marlize Bekker, email [m.bekker@uq.edu.au](mailto:m.bekker@uq.edu.au)

| **Table S1. Identification and relative abundance of volatile compounds detected in cacao nibs. Compound identification was based on agreement of multiple reaction monitoring (MRM) transitions, retention indices, standard retention times, and measured retention times. Relative abundance is expressed as percentage of total normalised peak area (mean ± standard deviation, n = 3).** | | | | | | | | | | | | | | | |
| --- | --- | --- | --- | --- | --- | --- | --- | --- | --- | --- | --- | --- | --- | --- | --- |
|  | **Method of Compound Identification** | | | | **Mount Edna** | | |  | **Fishery Falls** | | |  | **Shannon Vale** | | |
|  | **MRM^1^ transition** | **Measured RT^2^** | **Standard**  **RT** | **Retention Index** | **Percentage peak area** | | |  | **Percentage peak area** | | |  | **Percentage peak area** | | |
| Acetic acid |  |  |  |  | 66.243% | ± | 1.435% |  | 58.154% | ± | 1.304% |  | 77.774% | ± | 1.019% |
| 2-Phenylethanol | 91.00>65.00 | 11.50 | 11.47 | 1111 | 4.315% | ± | 0.791% |  | 7.378% | ± | 0.219% |  | 2.161% | ± | 0.171% |
| Benzaldehyde | 105.00>77.00 | 8.17 | 8.18 | 962 | 6.039% | ± | 0.398% |  | 5.952% | ± | 0.178% |  | 1.605% | ± | 0.072% |
| Isobutyl acetate |  |  |  |  | 2.853% | ± | 0.353% |  | 4.540% | ± | 0.119% |  | 1.243% | ± | 0.012% |
| 2,3-Butanediol |  |  |  |  | 3.326% | ± | 1.082% |  | 2.769% | ± | 0.121% |  | 3.210% | ± | 0.184% |
| 2-Phenylethyl acetate | 104.00>78.10 | 13.98 | 13.95 | 1254 | 1.820% | ± | 0.465% |  | 2.468% | ± | 0.139% |  | 1.549% | ± | 0.194% |
| 1-Penten-3-ol |  |  |  |  | 1.533% | ± | 0.637% |  | 1.702% | ± | 0.556% |  | 0.523% | ± | 0.027% |
| Ethyl acetate |  |  |  |  | 2.403% | ± | 0.086% |  | 2.128% | ± | 0.075% |  | 2.827% | ± | 0.113% |
| 2-Nonanol | 69.10>41.10 | 11.30 | 11.32 | 1101 | 0.248% | ± | 0.025% |  | 1.629% | ± | 0.068% |  | 0.163% | ± | 0.009% |
| Isobutyric acid | 73.10>55.00 | 2.91 | 2.81 | 743 | 0.735% | ± | 0.073% |  | 1.074% | ± | 0.086% |  | 0.291% | ± | 0.015% |
| 2-Nonanone | 71.00>43.00 | 11.08 | 11.09 | 1090 | 0.104% | ± | 0.018% |  | 1.008% | ± | 0.098% |  | 0.073% | ± | 0.001% |
| 2-Heptanol | 83.10>55.10 | 6.58 | 6.59 | 901 | 0.283% | ± | 0.034% |  | 0.848% | ± | 0.103% |  | 0.117% | ± | 0.003% |
| Isoamyl acetate | 70.10>55.10 | 5.84 | 5.67 | 875 | 0.849% | ± | 0.195% |  | 0.693% | ± | 0.040% |  | 1.457% | ± | 0.030% |
| 2-Methyl butyric acid | 74.00>56.00 | 5.11 | 4.92 | 852 | 0.473% | ± | 0.030% |  | 0.692% | ± | 0.019% |  | 0.164% | ± | 0.001% |
| 2-Methyl-1-butanol | 70.10>55.10 | 2.63 | 2.60 | 728 | 0.511% | ± | 0.066% |  | 0.623% | ± | 0.057% |  | 0.469% | ± | 0.013% |
| Isoamyl alcohol | 70.10>55.10 | 2.63 | 2.61 | 725 | 0.513% | ± | 0.064% |  | 0.626% | ± | 0.050% |  | 0.468% | ± | 0.015% |
| Acetophenone | 105.00>77.00 | 10.57 | 10.61 | 1066 | 0.729% | ± | 0.040% |  | 0.595% | ± | 0.018% |  | 0.375% | ± | 0.043% |
| 2-Acetylpyrrole | 109.10>94.10 | 10.51 | 10.44 | 1061 | 0.386% | ± | 0.104% |  | 0.462% | ± | 0.026% |  | 0.594% | ± | 0.059% |
| 2-Methylbutanal |  |  |  |  | 0.387% | ± | 0.107% |  | 0.391% | ± | 0.062% |  | 0.173% | ± | 0.009% |
| 2-Methylbutyl acetate | 70.00>55.10 | 5.84 | 5.87 | 877 | 0.567% | ± | 0.117% |  | 0.464% | ± | 0.037% |  | 0.760% | ± | 0.023% |
| 2,3-Dimethylpyrazine | 108.00>67.00 | 6.95 | 6.89 | 915 | 0.555% | ± | 0.042% |  | 0.456% | ± | 0.018% |  | 0.179% | ± | 0.004% |
| Methyl isobutyrate | 71.10>43.10 | 2.14 | 2.07 | 690 | 0.309% | ± | 0.087% |  | 0.323% | ± | 0.065% |  | 0.135% | ± | 0.008% |
| Phenylacetaldehyde | 91.10>65.10 | 10.09 | 10.07 | 1044 | 0.336% | ± | 0.027% |  | 0.393% | ± | 0.008% |  | 0.235% | ± | 0.017% |
| 2,3,5-Trimethylpyrazine | 122.10>42.10 | 9.20 | 9.06 | 997 | 0.538% | ± | 0.011% |  | 0.409% | ± | 0.023% |  | 0.229% | ± | 0.009% |
| 2-Heptanone | 71.00>43.00 | 6.21 | 6.26 | 888 | 0.095% | ± | 0.017% |  | 0.296% | ± | 0.027% |  | 0.038% | ± | 0.001% |
| Myrcene | 93.00>77.10 | 8.89 | 8.87 | 990 | 0.222% | ± | 0.020% |  | 0.294% | ± | 0.014% |  | 0.040% | ± | 0.023% |
| (Z)-β-Ocimene |  |  |  |  | 0.176% | ± | 0.024% |  | 0.233% | ± | 0.019% |  | 0.041% | ± | 0.030% |
| Isopropyl butyrate | 71.10>43.10 | 4.46 | 4.62 | 835 | 0.114% | ± | 0.032% |  | 0.226% | ± | 0.009% |  | 0.038% | ± | 0.003% |
| *trans*-2-Hexenal | 69.10>41.10 | 4.93 | 5.11 | 848 | 0.166% | ± | 0.009% |  | 0.204% | ± | 0.005% |  | 0.089% | ± | 0.002% |
| Ethyl 2-mercaptopropionate | 88.00>60.00 | 7.26 | 7.13 | 924 | 0.358% | ± | 0.016% |  | 0.186% | ± | 0.012% |  | 0.608% | ± | 0.035% |
| 2-Butanone |  |  |  |  | 0.133% | ± | 0.019% |  | 0.162% | ± | 0.022% |  | 0.080% | ± | 0.001% |
| Ethyl phenylacetate | 91.10>65.10 | 13.78 | 13.79 | 1242 | 0.134% | ± | 0.033% |  | 0.148% | ± | 0.008% |  | 0.150% | ± | 0.021% |
| Nonanal | 82.10>67.10 | 11.35 | 11.47 | 1104 | 0.016% | ± | 0.003% |  | 0.134% | ± | 0.007% |  | 0.010% | ± | 0.001% |
| 2,3-Butanedione |  |  |  |  | 0.177% | ± | 0.010% |  | 0.143% | ± | 0.004% |  | 0.062% | ± | 0.001% |
| δ-Terpinene |  |  |  |  | 0.256% | ± | 0.004% |  | 0.158% | ± | 0.019% |  | 0.078% | ± | 0.002% |
| Butyric acid | 60.00>42.00 | 3.31 | 3.17 | 769 | 0.163% | ± | 0.021% |  | 0.138% | ± | 0.010% |  | 0.121% | ± | 0.004% |
| Ethyl octanoate | 88.10>61.00 | 13.00 | 12.99 | 1195 | 0.089% | ± | 0.032% |  | 0.100% | ± | 0.022% |  | 0.228% | ± | 0.020% |
| Ethyl benzoate | 105.00>77.10 | 12.58 | 12.57 | 1171 | 0.126% | ± | 0.011% |  | 0.124% | ± | 0.004% |  | 0.102% | ± | 0.005% |
| 5-Ethyl-2,3-dimethylpyrazine | 135.10>53.10 | 10.96 | 10.93 | 1084 | 0.194% | ± | 0.003% |  | 0.127% | ± | 0.011% |  | 0.084% | ± | 0.009% |
| Propyl propionate | 75.10>57.10 | 3.49 | 3.78 | 806 | 0.120% | ± | 0.011% |  | 0.106% | ± | 0.003% |  | 0.066% | ± | 0.003% |
| (*E*)-Linalool oxide | 94.10>79.10 | 11.02 | 10.99 | 1087 | 0.077% | ± | 0.008% |  | 0.099% | ± | 0.005% |  | 0.034% | ± | 0.003% |
| 1-Hexen-3-ol | 57.10>31.10 | 3.31 | 3.32 | 771 | 0.104% | ± | 0.036% |  | 0.086% | ± | 0.005% |  | 0.104% | ± | 0.008% |
| 2-Methylpyrazine | 94.00>67.00 | 4.33 | 4.13 | 818 | 0.058% | ± | 0.002% |  | 0.077% | ± | 0.008% |  | 0.085% | ± | 0.006% |
| *trans*-2-Penten-1-ol | 57.10>31.00 | 3.31 | 3.09 | 763 | 0.094% | ± | 0.035% |  | 0.077% | ± | 0.003% |  | 0.097% | ± | 0.008% |
| 2,5-Dimethylpyrazine | 108.10>42.10 | 6.95 | 6.73 | 909 | 0.053% | ± | 0.001% |  | 0.064% | ± | 0.002% |  | 0.104% | ± | 0.008% |
| 3-Hexanone | 71.10>43.10 | 3.31 | 3.29 | 777 | 0.083% | ± | 0.025% |  | 0.064% | ± | 0.003% |  | 0.080% | ± | 0.005% |
| Pantolactone | 71.10>43.10 | 9.90 | 9.81 | 1037 | 0.030% | ± | 0.003% |  | 0.058% | ± | 0.000% |  | 0.021% | ± | 0.001% |
| Propanoic acid | 74.00>56.00 | 2.13 | 2.02 | 686 | 0.056% | ± | 0.002% |  | 0.056% | ± | 0.003% |  | 0.055% | ± | 0.001% |
| Acetoin | 45.00>27.00 | 2.25 | 2.23 | 705 | 0.069% | ± | 0.004% |  | 0.043% | ± | 0.012% |  | 0.047% | ± | 0.006% |
| 2-Pentanol | 73.10>55.10 | 2.25 | 2.20 | 699 | 0.079% | ± | 0.012% |  | 0.050% | ± | 0.004% |  | 0.054% | ± | 0.002% |
| Ethyl 3-methylbutanoate | 85.10>57.10 | 5.13 | 5.13 | 850 | 0.042% | ± | 0.003% |  | 0.049% | ± | 0.002% |  | 0.015% | ± | 0.006% |
| 2,6-Dimethylpyrazine | 108.00>42.00 | 6.95 | 6.76 | 910 | 0.051% | ± | 0.004% |  | 0.043% | ± | 0.001% |  | 0.025% | ± | 0.001% |
| ɣ-Heptalactone |  |  |  |  | 0.030% | ± | 0.005% |  | 0.027% | ± | 0.003% |  | 0.007% | ± | 0.002% |
| Epoxylinalool isomer-1 | 94.10>79.10 | 12.67 | 12.56 | 1171 | 0.026% | ± | 0.004% |  | 0.032% | ± | 0.003% |  | 0.010% | ± | 0.001% |
| Epoxylinalool isomer-2 | 94.10>79.10 | 12.67 | 12.65 | 1176 | 0.022% | ± | 0.005% |  | 0.029% | ± | 0.004% |  | 0.008% | ± | 0.000% |
| Ethyl hexanoate | 88.00>61.00 | 9.11 | 9.12 | 998 | 0.030% | ± | 0.002% |  | 0.027% | ± | 0.001% |  | 0.098% | ± | 0.003% |
| Ethyl 2-methylbutyrate | 102.00>74.00 | 4.98 | 4.99 | 845 | 0.033% | ± | 0.006% |  | 0.031% | ± | 0.005% |  | 0.014% | ± | 0.001% |
| 1-Hexen-3-one | 70.10>55.00 | 3.09 | 3.12 | 765 | 0.014% | ± | 0.005% |  | 0.024% | ± | 0.003% |  | 0.010% | ± | 0.001% |
| Capronic acid | 73.00>55.00 | 8.64 | 8.61 | 982 | 0.021% | ± | 0.004% |  | 0.026% | ± | 0.001% |  | 0.020% | ± | 0.003% |
| (E)-β-Ocimene |  |  |  |  | 0.025% | ± | 0.005% |  | 0.031% | ± | 0.006% |  | 0.009% | ± | 0.007% |
| Ethyl decanoate | 101.10>73.10 | 16.07 | 16.07 | 1393 | 0.011% | ± | 0.007% |  | 0.018% | ± | 0.007% |  | 0.032% | ± | 0.009% |
| Furfuryl alcohol | 98.10>42.10 | 5.11 | 5.12 | 848 | 0.014% | ± | 0.001% |  | 0.020% | ± | 0.001% |  | 0.089% | ± | 0.003% |
| (Z)-Linalool oxide | 94.10>79.10 | 10.70 | 10.75 | 1072 | 0.014% | ± | 0.001% |  | 0.020% | ± | 0.000% |  | 0.008% | ± | 0.000% |
| *trans*-Cinnamaldehyde | 103.10>77.10 | 14.23 | 14.28 | 1274 | 0.023% | ± | 0.005% |  | 0.022% | ± | 0.003% |  | 0.014% | ± | 0.002% |
| 2-Pentylfuran | 81.00>53.00 | 8.90 | 8.87 | 990 | 0.016% | ± | 0.002% |  | 0.019% | ± | 0.002% |  | 0.012% | ± | 0.001% |
| Furfural | 95.00>67.10 | 4.44 | 4.46 | 827 | 0.015% | ± | 0.002% |  | 0.018% | ± | 0.001% |  | 0.018% | ± | 0.003% |
| 3-Octanone | 71.10>43.10 | 8.78 | 8.77 | 986 | 0.012% | ± | 0.001% |  | 0.017% | ± | 0.001% |  | 0.008% | ± | 0.001% |
| 2-Ethyl-3,6-dimethylpyrazine | 135.10>107.10 | 10.83 | 10.73 | 1075 | 0.010% | ± | 0.003% |  | 0.013% | ± | 0.003% |  | 0.041% | ± | 0.006% |
| 3-Pentanone | 86.10>57.10 | 2.14 | 2.22 | 697 | 0.019% | ± | 0.005% |  | 0.015% | ± | 0.005% |  | 0.009% | ± | 0.001% |
| 2-Ethyl-5-methylpyrazine | 121.10>66.10 | 9.13 | 9.00 | 995 | 0.011% | ± | 0.001% |  | 0.011% | ± | 0.001% |  | 0.025% | ± | 0.002% |
| 2-Pentanone | 86.10>71.10 | 2.14 | 2.13 | 692 | 0.013% | ± | 0.004% |  | 0.010% | ± | 0.002% |  | 0.009% | ± | 0.000% |
| 2-Ethyl-3-methylpyrazine | 121.00>93.10 | 9.13 | 9.08 | 998 | 0.011% | ± | 0.001% |  | 0.011% | ± | 0.001% |  | 0.025% | ± | 0.003% |
| 2-Acetylfuran | 110.10>95.00 | 6.76 | 6.65 | 906 | 0.010% | ± | 0.000% |  | 0.011% | ± | 0.001% |  | 0.044% | ± | 0.004% |
| Benzyl acetate | 150.10>108.10 | 12.42 | 12.39 | 1162 | 0.013% | ± | 0.004% |  | 0.013% | ± | 0.002% |  | 0.008% | ± | 0.001% |
| Methyl isovalerate | 74.10>43.00 | 3.15 | 3.16 | 768 | 0.009% | ± | 0.002% |  | 0.011% | ± | 0.001% |  | 0.008% | ± | 0.000% |
| Ethyl butanoate | 71.00>43.10 | 3.66 | 3.61 | 800 | 0.022% | ± | 0.010% |  | 0.016% | ± | 0.006% |  | 0.016% | ± | 0.001% |
| 2-Ethylpyrazine | 107.00>79.00 | 7.01 | 6.84 | 913 | 0.008% | ± | 0.001% |  | 0.008% | ± | 0.002% |  | 0.012% | ± | 0.001% |
| Octanal | 84.00>55.00 | 9.16 | 9.23 | 1002 | 0.009% | ± | 0.005% |  | 0.011% | ± | 0.004% |  | 0.005% | ± | 0.000% |
| 1-Heptanol | 70.10>55.10 | 8.42 | 8.47 | 972 | 0.007% | ± | 0.001% |  | 0.007% | ± | 0.001% |  | 0.011% | ± | 0.004% |
| 3-Methyl-2-buten-1-ol | 71.10>43.10 | 3.15 | 2.93 | 764 | 0.012% | ± | 0.003% |  | 0.010% | ± | 0.002% |  | 0.012% | ± | 0.001% |
| Ethyl laurate | 88.10>61.00 | 18.73 | 18.74 | 1592 | 0.004% | ± | 0.001% |  | 0.007% | ± | 0.001% |  | 0.005% | ± | 0.002% |
| Indole | 117.00>90.00 | 14.63 | 14.60 | 1294 | 0.004% | ± | 0.001% |  | 0.007% | ± | 0.000% |  | 0.005% | ± | 0.001% |
| Isoamyl butyrate | 70.10>55.10 | 10.33 | 10.35 | 1057 | 0.009% | ± | 0.002% |  | 0.009% | ± | 0.002% |  | 0.005% | ± | 0.001% |
| Pyrazine |  |  |  |  | 0.003% | ± | 0.000% |  | 0.004% | ± | 0.001% |  | 0.001% | ± | 0.000% |
| 6-Methyl-5-hepten-2-one | 108.10>93.10 | 8.78 | 8.80 | 985 | 0.003% | ± | 0.000% |  | 0.004% | ± | 0.001% |  | 0.002% | ± | 0.000% |
| *trans*-2-Octenal | 83.10>55.10 | 10.37 | 10.37 | 1058 | 0.003% | ± | 0.001% |  | 0.004% | ± | 0.000% |  | 0.002% | ± | 0.000% |
| 3-Pentanol | 59.10>31.10 | 2.32 | 2.11 | 700 | 0.003% | ± | 0.000% |  | 0.003% | ± | 0.000% |  | 0.003% | ± | 0.001% |
